# Supplementary material for: Complexome profiling of the Chlamydomonas psb28 mutant reveals TEF5 as an early PSII assembly factor
Source: Plant Cell. 2025 Mar 17;37(6):koaf055. doi: 10.1093/plcell/koaf055 (PMC12164590; doi:10.1093/plcell/koaf055)
Supplement: koaf055_Supplementary_Data [file koaf055_supplementary_data.zip › psb28_interactive.zip!/plots/intensity/qualityPlots/peptides/psbE_Cre-1.g2716990.t1.p1.html]

### Cre-1.g2716990.t1.p1

Trivial name: psbE  
SeqCoverage [median]: 53.70  
Mass: 9.30 kDa  
  
 

| Sequence | MissedCleavage | Mass [Da] | wtIntensity | muIntensity | Score |
| --- | --- | --- | --- | --- | --- |
| AGKPVERPFSDILTSIR | 2 | 1885.04 | 108896.95 | 7946.75 | 93.50 |
| FNALEQVK | 0 | 947.51 | 1159129.59 | 168248.51 | 233.21 |
| FNALEQVKK | 1 | 1075.60 | 429485.60 | 101538.92 | 162.97 |
| PFSDILTSIR | 0 | 1147.62 | 31750.57 | 0.00 | 179.51 |
| PNEYFTEDR | 0 | 1169.50 | 214159.12 | 17947.34 | 290.87 |
| PNEYFTEDRQEAPLITDR | 1 | 2193.03 | 11272.49 | 2192.12 | 97.90 |
| PVERPFSDILTSIR | 1 | 1628.89 | 34259.65 | 4321.52 | 148.57 |
| QEAPLITDR | 0 | 1041.55 | 1413481.48 | 148489.44 | 272.66 |
| QEAPLITDRFNALEQVK | 1 | 1971.04 | 270015.15 | 70667.91 | 190.82 |
| QEAPLITDRFNALEQVKK | 2 | 2099.14 | 258823.32 | 53960.69 | 190.46 |
|  |  |  | 3931273.9 | 575313.2 |  |
